# Treatment and outcomes in patients with non-TB mycobacterial pulmonary disease: a single-centre study

**DOI:** 10.5588/ijtldopen.25.0379

**Published:** 2026-01-09

**Authors:** V.N. Dahl, A. Fløe, F. Rudolf, J. van Ingen, A.B. Andersen, T. Lillebaek, C.M. Wejse

**Affiliations:** 1 Department of Infectious Diseases, Aarhus University Hospital, Aarhus, Denmark;; 2 Center for Global Health, Department of Public Health, Aarhus University (GloHAU), Aarhus, Denmark;; 3 International Reference Laboratory of Mycobacteriology, Statens Serum Institut, Copenhagen, Denmark;; 4 Department of Respiratory Diseases and Allergy, Aarhus University Hospital, Aarhus, Denmark;; 5 Department of Medical Microbiology, Radboud University Medical Center, Nijmegen, the Netherlands;; 6 Department of Infectious Diseases, Rigshospitalet, Copenhagen, Denmark;; 7 Global Health Section, Department of Public Health, University of Copenhagen, Copenhagen, Denmark.

**Keywords:** tuberculosis, NTM, Denmark, pulmonary infection, treatment, outcomes, mortality

## Abstract

**BACKGROUND:**

In Denmark, information on treatment and outcomes of non-TB mycobacterial pulmonary disease (NTM-PD) are limited. We aimed to evaluate treatment and clinical outcomes in patients with pulmonary NTM isolates.

**METHODS:**

We conducted a retrospective cohort study of all patients with pulmonary NTM isolates, except *Mycobacterium gordonae* alone, from 2016 to 2021 in the Central Region of Denmark. Clinical data were manually extracted from hospital records. Treatment outcomes (NTM-NET definitions) were assessed in treated patients, while culture conversion and mortality were evaluated in all patients.

**RESULTS:**

Among 164 patients (median age 70 years; 48.1% male), 54.3% received antibiotic therapy, and 30% of patients meeting diagnostic criteria were not treated. Most treated patients (87.6%) were prescribed an ethambutol–macrolide–rifamycin regimen; few received intravenous (*n* = 1) or second-line drugs (*n* = 14), despite high rates of cavitary disease (>50%). Only 53.9% achieved a favourable outcome. Culture conversion within 12 months of the first positive NTM culture was associated with lower all-cause mortality (*P* < 0.001). All-cause mortality was 11% at 1 year and 25% at 3 years.

**CONCLUSION:**

To improve outcomes for NTM-PD patients, improved microbiological monitoring, guideline adherence, individualised therapy for high-risk patients, consideration of prolonged or intensified therapy, and better treatment regimens are needed.

Treatment of non-TB mycobacterial pulmonary disease (NTM-PD) is challenging, often requiring prolonged multidrug antibiotic regimens.^1^ These regimens, which are often associated with significant adverse effects, typically span months to years, yet frequently lead to suboptimal results.^1–3^ While guidelines for NTM-PD treatment are available,^1,4^ adherence to these recommendations is inconsistent.^5–7^ In Denmark, there are limited data on treatment practices and clinical outcomes for patients with NTM-PD. Only larger population-based studies on NTM-PD-related mortality from Denmark exist,^8,9^ but they lack detailed clinical information. To address this gap, we evaluated treatment and clinical outcomes among patients with NTM isolated from the lungs.

## METHODS

We conducted a retrospective cohort study in the Central Region of Denmark (approx. 1.2 million inhabitants) from 2016 through 2021, including all patients with NTM isolated from the lungs, excluding those with *Mycobacterium gordonae* only. The study was based on data from a previously described cohort.^10^ NTM species were identified at the centralised International Reference Laboratory of Mycobacteriology (IRLM), Statens Serum Institut, Copenhagen.^11^ The study setting and laboratory methods are described in detail elsewhere.^10,11^

Clinical information was manually extracted from the electronic hospital records. Data collected included comorbidities, risk factors for NTM disease and a poor outcome, lung function test results, imaging findings, and microbiological and biochemical results.^10^ Diagnostic criteria for NTM-PD were assessed according to international guidelines.^1^ Detailed treatment information was collected for patients treated with antibiotics for NTM-PD, including outcomes based on definitions from an NTM-NET consensus statement.^12^ As a less stringent outcome measure, any culture conversion (i.e., the absence of a subsequent positive culture, regardless of the number of samples) was assessed within 6 and 12 months after the first positive isolate. All-cause mortality data were collected for all patients.

Descriptive statistics were used to summarise the cohort, with categorical variables presented as numbers with percentages and continuous variables as medians with interquartile ranges (IQRs). Differences in all-cause mortality by 1) diagnostic criteria, 2) antibiotic therapy, 3) culture conversion within 12 months, and 4) BACES scores were illustrated using cumulative all-cause mortality plots and compared with log-rank tests. The BACES score is a validated score that aims to predict all-cause mortality in patients with NTM-PD.^13^ It is composed of body mass index < 18.5 kg/m^2^, age ≥ 65 years, presence of cavity on computed tomography, elevated erythrocyte sedimentation rate (>15 mm/h in men and 20 mm/h in women), and male sex, each of which gives one point. All analyses were performed using R (v. 4.2.3).

### Ethical statement

Ethical approval was granted by local authorities in the Central Denmark Region (1-16-5-72-719-23).

## RESULTS

From 2016 to 2021, 164 patients had NTM isolated from the lungs, excluding 29 patients with *M. gordonae* alone. Baseline characteristics of study participants are presented in Table 1. Of the 164 patients, 89 (54.3%) were treated for NTM-PD, with 71 (80%) meeting diagnostic criteria. Among 101 patients meeting the diagnostic criteria, 29.7% (*n* = 30) were not treated. The proportion of treated patients varied by species: *M. malmoense* (100%, *n* = 2/2), *M. abscessus* (83.3%, *n* = 5/6), *M. kansasii* (66.7%, *n* = 2/3), *M. avium* complex (62%, *n* = 67/108), *M. xenopi* (38.1%, *n* = 8/21), *M. szulgai* (33.3%, *n* = 1/3), and *M. chelonae* (0%, *n* = 0/6). Most treated patients (87.6%, *n* = 78/89) were prescribed an ethambutol–macrolide–rifamycin regimen (Table 2), with 51.3% (*n* = 40) administered azithromycin as the macrolide component. Only a smaller number of patients received other drugs, including isoniazid (*n* = 5), fluoroquinolones (*n* = 3), doxycycline (*n* = 2), inhaled liposomal amikacin (*n* = 2), linezolid (*n* = 2), clofazimine (*n* = 1), ethionamide (*n* = 1), inhaled amikacin suspension (*n* = 1), and intravenous amikacin (*n* = 1). Of those treated, 53.9% (*n* = 48/89) had favourable treatment outcomes, categorised as culture conversion, microbiological cure, or clinical cure, based on NTM-NET definitions. The remaining patients experienced unfavourable outcomes, including failure or relapse, treatment discontinuation, unknown outcomes, or death within 12 months of treatment initiation.

**Table 1.** Baseline characteristics for patients with NTM isolated from the lungs (*n* = 164).

Characteristics	
Age in years, median (IQR)	70 (17)
Male, *n*/*N* (%)	79/164 (48)
BMI (kg/m^2^), median (IQR)^**A**^	21.8 (6.0)
CCI score, *n*/*N* (%)	
0	26/164 (16)
1–2	108/164 (66)
3–4	18/164 (11)
>4	12/164 (7.3)
Comorbidities and risk factors, *n*/*N* (%)	
Current/previous smoking	120/161 (75)
COPD/emphysema	102/164 (62)
Use of ICS	70/164 (43)
Bronchiectasis	61/164 (37)
Proton pump inhibitor	49/164 (30)
Asthma	13/164 (7.9)
Immunosuppression^**B**^	19/164 (12)
Lung cancer	10/164 (6.1)
History of TB	5/164 (3.0)

NTM = non-TB mycobacteria; IQR = interquartile range; BMI = body mass index; CCI = Charlson Comorbidity Index; COPD = chronic obstructive pulmonary disease; ICS = inhaled corticosteroid.

A Missing values for 25 patients.

B One or more of the following: systemic steroids (>10 mg) (*n* = 8), disease-modifying anti-rheumatic drugs (*n* = 7), mycophenolate mofetil and/or tacrolimus (*n* = 3), TNF-alpha-inhibitor (*n* = 2), chemotherapy (*n* = 1) or radiation (*n* = 2), solid organ transplantation (*n* = 1), and rituximab (*n* = 1).

**Table 2.** Treatment and outcomes of patients treated for NTM pulmonary disease (*n* = 89).

Treatment	
Treatment duration, days, median (IQR)	398 (348)
Ethambutol–macrolide–rifamycin at start, *n*/*N* (%)	78/89 (88)
Macrolide for >8 weeks, *n*/*N* (%)	80/89 (90)
Dosing strategy, *n*/*N* (%)	
Daily	61/88 (69)
Thrice weekly	20/88 (23)
Change in strategy	7 (8.0)
More than three drugs used, *n*/*N* (%)	15/89 (17)
More than one change in drug regimen, *n*/*N* (%)^**A**^	6/89 (6.7)
Days of admission, median (IQR)^**B**^	9 (23)
Outpatient visits, median (IQR)	10.0 (5.0)
Outcome	
NTM-NET treatment outcomes, *n*/*N* (%)^**C**^	
Culture conversion	26/89 (29)
Microbiological cure	12/89 (13)
Clinical cure	22/89 (25)
Failure/relapse	21/89 (24)
Treatment halted	10/89 (11)
Unknown	2/89 (2.2)
Any culture conversion, *n*/*N* (%)	
<6 months	23/89 (26)
<12 months	38/89 (43)
Same species isolated 12 months after first isolation, *n*/*N* (%)	23/89 (26)
All-cause mortality, *n*/*N* (%)	
Dead within 12 months of first positive NTM culture	6/89 (6.7)
Dead within 12 months of treatment	8/89 (9.0)
Dead during follow-up	29/89 (33)

NTM = non-TB mycobacteria; IQR = interquartile range.

A 15 patients had one change of drug, 5 had two, and 1 had four changes while 68 patients did not have any drug changes.

B 38 patients were not admitted during diagnosis/treatment of NTM.

C According to a previous NTM-NET consensus statement.^12^

Across the entire cohort, the median follow-up time from the first positive sample to data collection was 56.5 months (IQR: 42.1–66.6). For all patients, including untreated patients, 61 (37.2%) died during a median follow-up of 41.9 months (IQR: 26.1–59.4). All-cause mortality rates at 6, 12, 24, and 36 months following the first positive NTM culture were 8.8% (*n* = 17), 11.4% (*n* = 22), 17.6% (*n* = 34), and 25.4% (*n* = 49), respectively. Cumulative all-cause mortality was not significantly different by diagnostic criteria or antibiotic therapy, although a trend toward lower all-cause mortality was observed in treated patients (Figure Panel A and B). In contrast, both culture conversion within 12 months of the first positive NTM culture (*P* < 0.001) and a lower BACES score (*P* = 0.0021) were associated with lower cumulative all-cause mortality (Figure Panel C and D). A stratified post hoc analysis showed that the proportions of culture conversion within 12 months were similar between treated (43%, *n* = 38/89) and untreated patients (40%, *n* = 30/75) (Pearson’s *χ*^2^ test, *P* = 0.7). Among those with at least one follow-up culture, culture conversion was observed in 48% (*n* = 30/63) of treated and 25% (*n* = 3/12) of untreated (*P* = 0.15).

**Figure.**
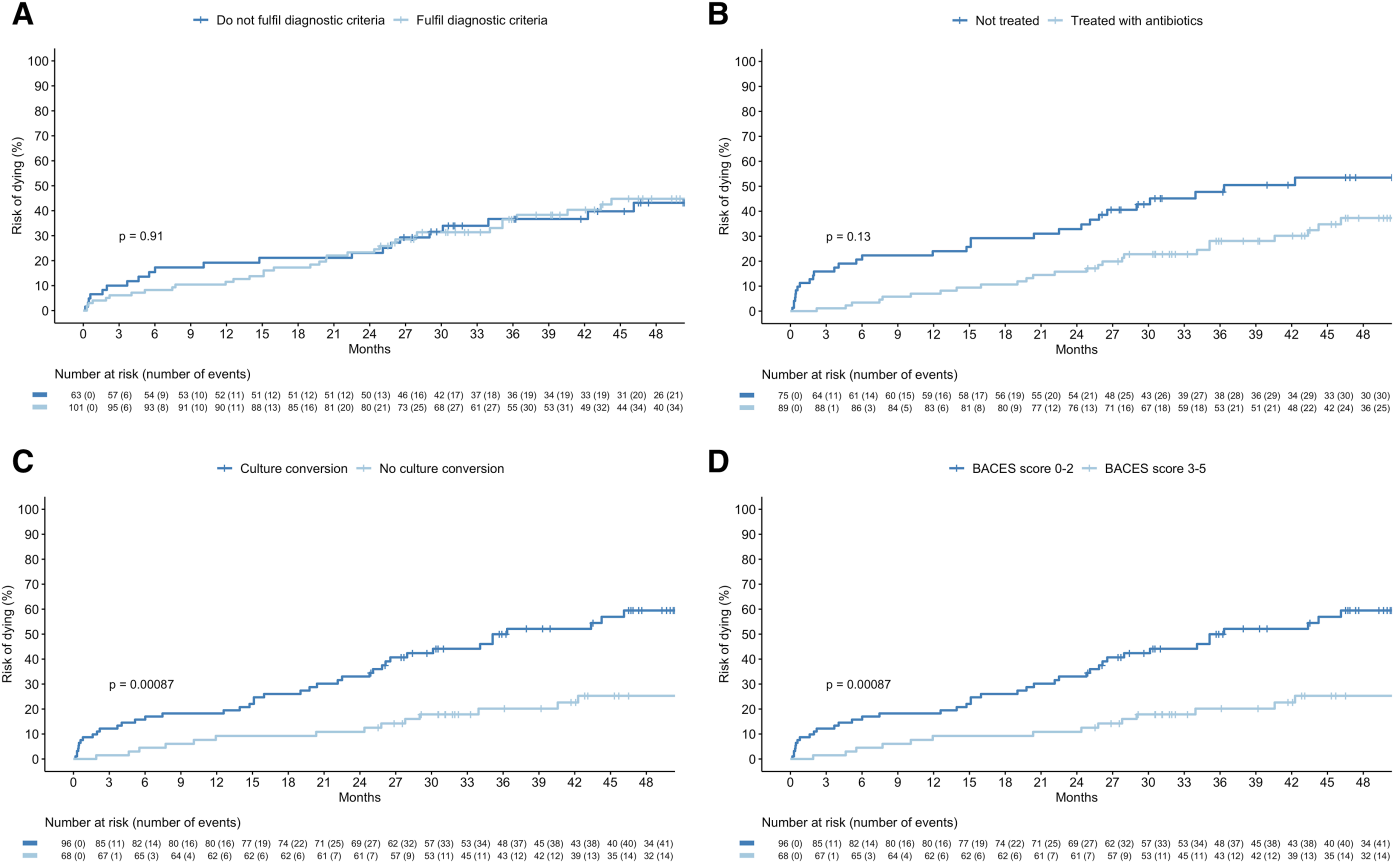
Cumulative all-cause mortality for patients with pulmonary NTM isolates (*n* = 164) by **A:** fulfilment of diagnostic criteria, **B:** treatment with antibiotics, **C:** any culture conversion within 12 months after first isolation of NTM, and **D:** BACES score. *P* values are derived from the log-rank test. When stratified by diagnostic criteria and treatment, mortality was lower among those treated, although it was not statistically significant (Supplementary Data Figure S2). Among all patients included in the BACES score analysis (*n* = 164), there were missing values for body mass index (*n* = 25), cavity (*n* = 15), and erythrocyte sedimentation rates (*n* = 130). The plot was comparable when based on an imputed dataset (Supplementary Data Figure S3A) and when replacing an elevated erythrocyte sedimentation rate with an elevated C-reactive protein (>7 mg/L) (see Supplementary Data Figure S3B).

As supplementary post hoc analyses, we explored adjusted predictors of all-cause mortality; hazard ratios from univariable and multivariable Cox regression analyses are presented in Supplementary Data.

## DISCUSSION

This study provides valuable insights into current treatment practices and outcomes of NTM-PD in a high-resource setting. About half of the patients with NTM isolated from the lungs were treated, and most of those treated fulfilled diagnostic criteria. However, nearly one third of patients meeting diagnostic criteria were not treated, reflecting the complexity of treatment decisions in this population. Although sputum culture conversion rates were similar between treated and untreated patients, this finding should be interpreted cautiously, given the likely selection of patients for treatment based on disease severity, comorbidities, and overall prognosis. All-cause mortality rates were substantial, with 11% and 25% of patients dying within 1 and 3 years of follow-up, respectively.

Despite the high prevalence of lung cavitation and many patients experiencing unfavourable outcomes, the use of intravenous therapy and drugs other than ethambutol, macrolides, and rifamycins was limited. Only one patient received intravenous amikacin. Although the exact additive effect of intravenous amikacin remains controversial, this low uptake in a population that included 56% (*n* = 50) treated cases of cavitary disease is remarkable.^14^ Additionally, one third (32%) of treated patients received therapy for less than 365 days, which falls short of the recommended treatment duration of 12 months beyond culture conversion for most species.^1,4^ More patients may have benefited from prolonged or intensified treatment, including intravenous or second-line drugs. A comparable study from the Netherlands observed that only 47% of NTM-PD patients remained on triple-drug therapy after 6 months, dropping to 19% after 1 year.^14^ The authors suggested that management could be improved through better adherence to guidelines and greater use of expert centres.

Consistent with current guidelines for *M. avium* complex and other species,^1,4^ many patients were prescribed an ethambutol–macrolide–rifamycin regimen. Clarithromycin was frequently used despite more recent recommendations favouring azithromycin.^1^ However, the proportion of azithromycin use increased from 31% in 2016 to 67% in 2021, suggesting that clinical practice is gradually aligning with the most recent guidelines. A recent study observed a four-fold increase in cure rates, and another an almost 20% lower hazard of 3-year all-cause mortality, among patients receiving guideline-based treatment compared to those who did not, underscoring the importance of guideline-based management.^15,16^ In our cohort, 64.6% of those with *M. avium* complex disease (*n* = 42/65, excluding two with unknown treatment duration) received treatment consistent with guideline recommendations, defined as a treatment duration exceeding 365 days, initiation with a macrolide, ethambutol, and a rifamycin, and daily dosing for cavitary disease. However, when considering aminoglycoside therapy as mandatory for guideline-based treatment of cavitary disease, only 26.2% of patients had guideline-adherent treatment. Only one of the five patients with *M. abscessus* received intravenous therapy as recommended in guidelines, but this patient was treated for only a little more than 3 months in total. Patients treated for *M. xenopi* were administered the recommended regimens, but none received amikacin despite seven out of eight having cavities. All except one were treated for more than a year. The two patients treated for *M. kansasii* received appropriate regimens daily for more than a year. These findings show that adherence to treatment principles were inconsistent, possibly reflecting factors such as limited awareness of guidelines, tolerability concerns, or practical challenges in implementation. At the same time, clearer and more specific guidance for managing complex or refractory cases may be helpful.

A nationwide population-based study of pulmonary NTM isolates in Denmark from 1997 to 2008 reported a cumulative 3-year all-cause mortality of 27.3%–33.6%, slightly higher than our estimate (25%).^9^ That study identified high levels of comorbidity, advanced age, male sex, and *M. xenopi* infection as negative prognostic factors. More recently, a nationwide study from Denmark using ICD-10 diagnosis codes for case finding reported a 3-year all-cause mortality of 29% among patients with presumed NTM-PD, based on data from 2000 to 2017.^8^ The differences in mortality rates are not substantial enough to be considered definite, and the estimates may not be directly comparable.

We observed no statistically significant difference in cumulative all-cause mortality rates between treated and untreated patients. A lack of association between first-line treatment and improved all-cause mortality has previously been reported.^17^ However, patients selected for treatment likely differed substantially from those who were not, with treatment decisions influenced by age, comorbidities, NTM species, disease severity, prognosis, other baseline patient characteristics, and perceived treatment benefit.^11,18^ The absence of robust natural history studies and randomised controlled trials in NTM-PD makes it challenging to determine the true impact of antibiotic therapy on survival. Without well-defined control groups and observational data based on highly selected populations, it remains challenging to distinguish the natural course of disease from comorbidities and potential treatment effects. Still, we observed a trend indicating that antibiotic therapy and culture conversion were associated with lower all-cause mortality, presumably influenced by limited statistical power. Our findings likely reflect generally poor treatment outcomes and suggest that treatment benefits may be confined to specific patient subgroups.

Culture conversion was associated with lower cumulative all-cause mortality, but no significant difference in culture conversion rates was observed between treated and untreated patients. This again suggests that factors beyond antibiotic therapy, such as host immune status and pathogen virulence, are critical to treatment outcomes.^19^ Supporting this, studies on the natural history of NTM-PD have shown that spontaneous culture conversion may occur in up to 60% of cases and radiological improvement in up to 50%.^20^ In our study, follow-up cultures were not available for many patients, and the treated and untreated groups included individuals who both met and did not meet diagnostic criteria. Only 20% (*n* = 18) of treated patients in our cohort had only one follow-up sample collected during treatment, and 29% (*n* = 26) had no samples, indicating a need for more rigorous monitoring of microbiological treatment response. In addition, some untreated patients may have been deemed unsuitable for treatment due to advanced disease or poor prognosis, potentially confounding this comparison. Despite our findings, multiple studies have consistently demonstrated an association between culture conversion and improved outcomes, underlining its usefulness as a biomarker for treatment response.^21–23^ A recent study found that 41% of patients had spontaneous culture conversion,^15^ comparable to the 43% conversion rate observed among treated patients in our cohort. Again, this underscores the complex interplay between host factors, pathogen, and treatment efficacy. Overall, we observed lower treatment success rates than those reported in similar recent studies,^15,24^ supporting that a more aggressive therapeutic strategy might have benefited some patients.

This study has several limitations. Its retrospective design may introduce misclassification bias due to incomplete or inaccurate data, particularly regarding disease severity, treatment adherence, and outcomes. For instance, culture conversion was not consistently documented throughout treatment for all patients. Treatment decisions were not randomised, introducing potential selection bias. Additionally, only one author evaluated and extracted patient data, which could have introduced observer bias. Lastly, the sample size limited our ability to assess the impact and outcomes associated with specific NTM species. Despite these limitations, our study, by presenting 5 years of real-world data, provides valuable insights into the current treatment practices for NTM-PD in a high-resource setting.

## CONCLUSION

This study demonstrates the challenges of managing NTM-PD, including variability in treatment practices, suboptimal cure rates, and substantial all-cause mortality. Our findings show the need for improved adherence to treatment guidelines, more rigorous microbiological monitoring during and after treatment, consideration of prolonged or intensified therapy for selected patient subgroups, and the need to optimise treatment regimens.

## Supplementary Material




