# Supplementary material for: Treatment and outcomes in patients with non-TB mycobacterial pulmonary disease: a single-centre study
Source: IJTLD Open. 2026 Jan 9;3(1):11–6. doi: 10.5588/ijtldopen.25.0379 (PMC12810752; doi:10.5588/ijtldopen.25.0379)
Supplement: Supplementary file 1 [file ijtldopen25-0379_supplementarydata1.pdf]

Supplementary to "*Treatment and outcomes in patients with nontuberculous mycobacterial pulmonary disease: a single-centre study*" by Dahl et al.

Supplementary 1. Predictors of mortality

A Cox proportional hazards regression was used to evaluate predictors of all-cause mortality in univariable and multivariable analyses. Predictors for the univariable model were selected based on previous studies and clinical relevance (1-3). Given the limited number of patients and events relative to candidate predictors, we refrained from building a manually selected multivariable model to avoid overfitting.

Four multivariable models were performed:

1. A model using stepwise backward selection with the Akaike Information Criterion (AIC) (R package MASS v. 7.3-60);
2. A model using Least Absolute Shrinkage and Selection Operator (Lasso) regularisation (R package glmnet v. 4.1-8), suitable for high-dimensional data and multicollinearity;
3. A model including only variables from the BACES score (body mass index <18.5 kg/m², age ≥65 years, lung cavity, elevated ESR, male sex) (3);
4. An alternative BACES model substituting CRP >7 mg/L for ESR.

These models are described in detail elsewhere (4). Proportional hazards assumptions were tested using Schoenfeld residuals and residual plots. Missing values for predictor variables were handled using multiple imputation by chained equations (MICE) (R package mice v. 3.16.0), with 100 imputations and 10 iterations under the assumption of missing at random (5). Dichotomous variables were imputed using logistic regression, categorical variables using polytomous regression, and continuous variables using predictive mean matching. Imputed values were evaluated against observed distributions to ensure plausibility. The distribution of imputed and observed values is shown in **Figure S1** and **Table S1**.

Hazard ratios from univariable and multivariable analyses are presented in **Table S2**. Across the models, variables consistently associated with higher all-cause mortality included BACES score components (except lung cavitation), comorbidity, severely to very severely reduced FEV1, elevated

CRP, and smear positivity. Conversely, antibiotic therapy and culture conversion showed a trend toward reduced mortality, although not statistically significant. A violation of the proportional hazards assumption was detected for antibiotic therapy (p = 0.0028). To address this, we performed a time-split Cox regression, fitting separate models for an early period (0-24 months) and a late period (24-48 months). In the early period, antibiotic therapy was associated with a 43% reduction in hazard (HR = 0.57; 95% CI: 0.28–1.16; p = 0.12), while in the late period, it was associated with an 11.8% increase in hazard (HR = 1.12; 95% CI: 0.52–2.42; p = 0.78), suggesting a time-varying treatment effect.

Our findings align with prior studies identifying similar mortality predictors (1, 3, 6). Both ESR and CRP have been independently associated with all-cause mortality in previous research (7, 8); however, no evidence favors one marker over the other. Our results support CRP as a potential alternative to ESR within the BACES score, though larger studies are needed to validate this substitution.

Figure S1. Missing predictor variables and variables of interest before imputation.

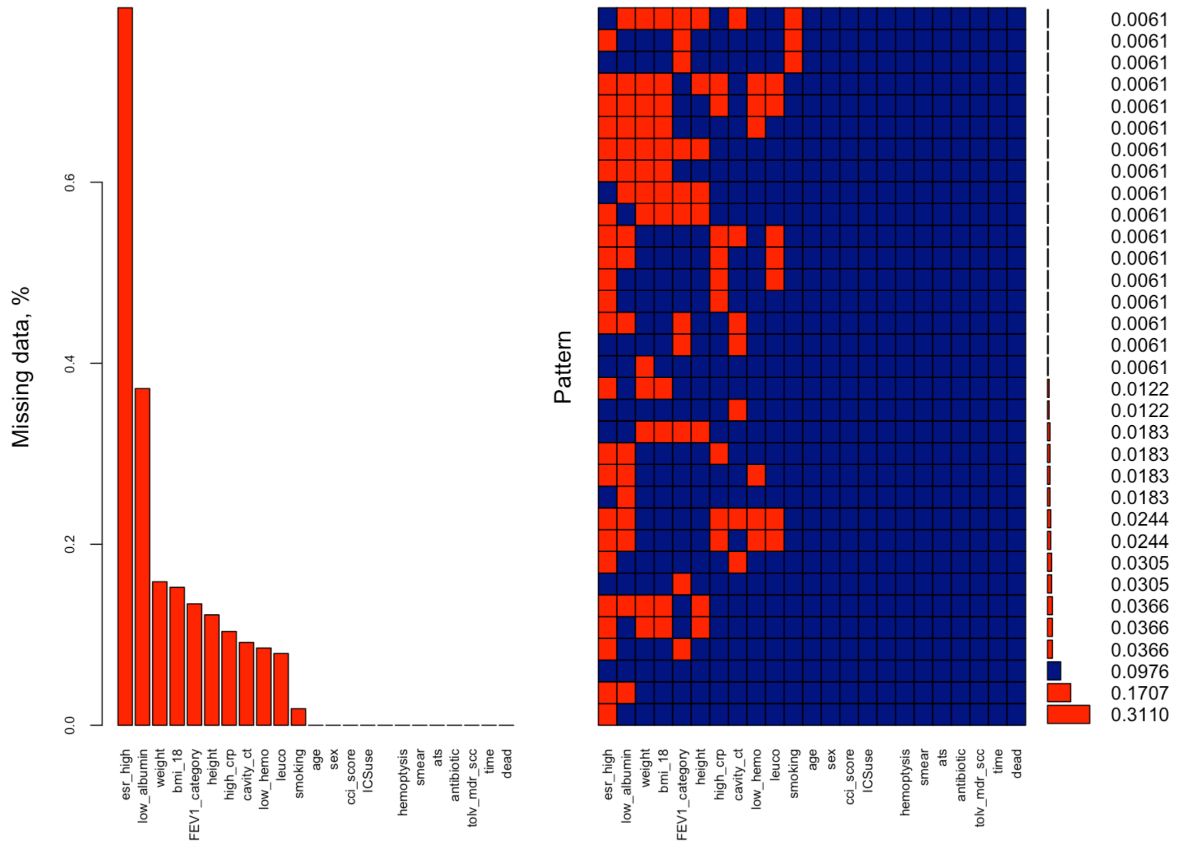

Table S1. Distribution of variables of interest before and after imputation.

Variable	Unimputed, n / N	Imputed, n / N	Missing values imputed, n
Current/previous smoking	120 / 161 (75)	122 / 164 (74)	3
BMI < 18.5 kg/m2	36 / 139 (26)	39 / 164 (24)	25
Elevated CRP (> 7 mg/L)	85 / 147 (58)	95 / 164 (58)	17
Anaemia (Hgb < 7 mmol/L)	19 / 150 (13)	22 / 164 (13)	14
Low albumin (< 30 g/L)	27 / 103 (26)	41 / 164 (25)	61
Elevated ESR (>15 mm/h males, > 20 mm/h females)	17 / 34 (50)	83 / 164 (51)	130
Any lung cavitation on CT/HRCT	77 / 149 (52)	85 / 164 (52)	15
FEV1 group			22
Normal	33 / 142 (23)	40 / 164 (24)	
Mild/moderate	58 / 142 (41)	64 / 164 (39)	
Severe/very severe	51 / 142 (36)	60 / 164 (37)	

Table S2. Cox proportional hazards model for all-cause mortality among patients with pulmonary NTM isolates (n=164).

Predictor	Univariable analysis			Multivariable analysis (Stepwise)*		Multivariable analysis (Lasso)**		Multivariable analysis (BACES score)***		Multivariable analysis (Alternative BACES score)****	
	n	HR (95%CI)	p-value	HR (95%CI)	p-value	HR (95%CI)	p-value	HR (95%CI)	p-value	HR (95%CI)	p-value
Older than 65 years	164	2.20 (1.24, 3.90)	0.007	1.95 (0.96, 3.95)	0.065	1.98 (0.98, 3.98)	0.057	2.96 (1.58, 5.56)	<0.001	3.43 (1.83, 6.42)	<0.001
Male	164	1.90 (1.14, 3.18)	0.014	1.73 (0.96, 3.12)	0.069	1.72 (0.95, 3.12)	0.073	1.94 (1.12, 3.37)	0.019	2.19 (1.28, 3.75)	0.004
Current/previous smoking	161	1.32 (0.70, 2.49)	0.4								
BMI < 18.5 kg/m2	139	2.10 (1.24, 3.54)	0.006	2.25 (1.14, 4.42)	0.019	2.16 (1.06, 4.40)	0.033	2.58 (1.48, 4.51)	<0.001	2.79 (1.63, 4.79)	<0.001
CCI group	164										
0		Reference		Reference							
1-2		1.68 (0.65, 4.30)	0.3	1.43 (0.48, 4.26)	0.5	Reference					
3-4		4.37 (1.54, 12.4)	0.006	4.57 (1.31, 15.9)	0.017	3.12 (1.32, 7.37)	0.009				
>4		9.17 (3.16, 26.6)	<0.001	28.3 (7.03, 114)	<0.001	17.1 (6.22, 46.9)	<0.001				
Inhaled corticosteroids	164	1.58 (0.95, 2.61)	0.075								
Immunosuppression	164	2.38 (1.26, 4.48)	0.007			1.20 (0.53, 2.69)	0.7				
Haemoptysis	164	0.50 (0.22, 1.17)	0.11								
FEV1 category	142										
Normal		Reference		Reference		Reference					
Mild/moderate		1.71 (0.67, 4.34)	0.3	0.49 (0.19, 1.31)	0.2	0.55 (0.22, 1.39)	0.2				
Severe/very severe		4.55 (1.89, 10.9)	<0.001	3.15 (1.18, 8.38)	0.022	3.22 (1.18, 8.78)	0.022				
Elevated CRP (> 7 mg/L)	147	2.75 (1.50, 5.05)	0.001							3.01 (1.63, 5.57)	<0.001
Elevated ESR (>15 mm/h males, > 20 mm/h females)	34	6.36 (1.39, 29.1)	0.017	12.4 (5.76, 26.5)	<0.001	11.0 (4.91, 24.6)	<0.001	4.67 (2.46, 8.87)	<0.001		
Anaemia (Hgb < 7 mmol/L)	150	3.06 (1.61, 5.82)	<0.001			1.00 (0.46, 2.19)	>0.9				
Low albumin (< 30 g/L)	103	3.66 (2.05, 6.55)	<0.001			1.22 (0.59, 2.49)	0.6				
Smear positivity	164	1.29 (0.72, 2.31)	0.4	2.41 (1.21, 4.80)	0.013	2.35 (1.17, 4.72)	0.017				
Lung cavitation on CT/HRCT	149	1.21 (0.71, 2.08)	0.5					1.08 (0.64, 1.81)	0.8	0.93 (0.55, 1.56)	0.8
Fulfilling diagnostic criteria	164	1.03 (0.61, 1.73)	0.9								
Antibiotic therapy	164	0.68 (0.41, 1.12)	0.13	0.61 (0.32, 1.16)	0.13	0.61 (0.32, 1.16)	0.13				
Any culture conversion < 12 months	164	0.39 (0.21, 0.69)	0.001	0.60 (0.31, 1.15)	0.12	0.55 (0.28, 1.08)	0.080				

Abbreviations: NTM, nontuberculous mycobacteria. HR, hazard ratio. CI, confidence interval. BMI, body mass index. CCI, Charlson comorbidity index. FEV1, forced expiratory volume in 1 second. CRP, C-reactive protein. ESR, erythrocyte sedimentation rate. Hgb, haemoglobin. CT/HRCT, computed tomography/high-resolution computed tomography.

*Stepwise backward selection with the Akaike Information Criterion (AIC) starts with a full model and iteratively removes predictors to minimize the AIC, balancing model fit and simplicity (4).

**Lasso is a regularization technique that minimizes the residual sum of squares with a penalty proportional to the sum of the absolute values of the coefficients, shrinking some to zero for automatic variable selection (4).

***The BACES score is a validated index predicting all-cause mortality in patients with NTM pulmonary disease, comprising BMI <18.5 kg/m², age ≥65 years, presence of cavity on CT, ESR, and male sex (3).

****In the alternative BACES model, CRP >7 mg/L was substituted for ESR.

Supplementary 2. Cumulative all-cause mortality by antibiotic treatment, diagnostic criteria, and BACES scores.

Figure S2. Cumulative all-cause mortality for patients with pulmonary NTM isolates (n=164) by those fulfilling diagnostic criteria (Panel A) and those who do not (Panel B), stratified by treatment with antibiotics.

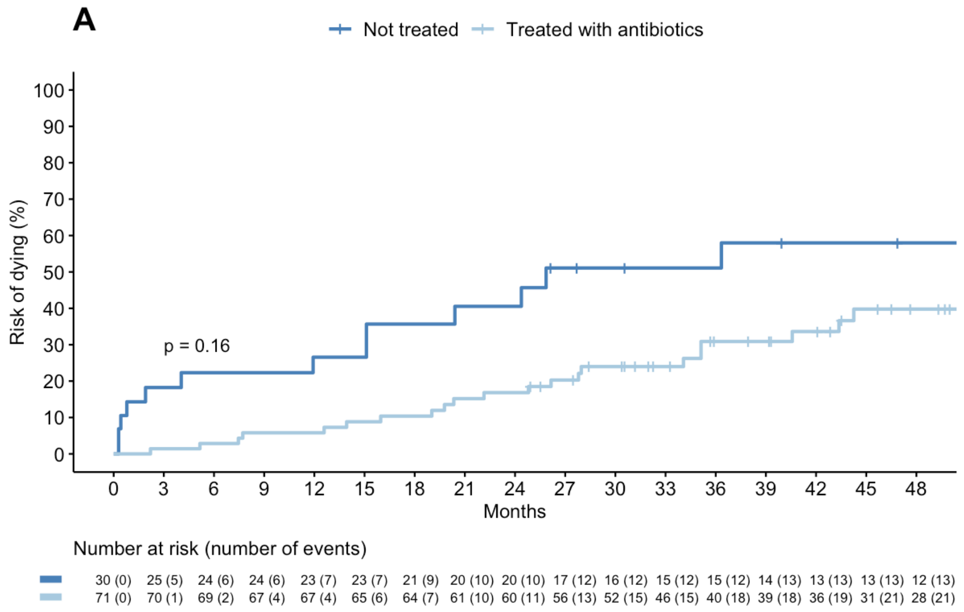

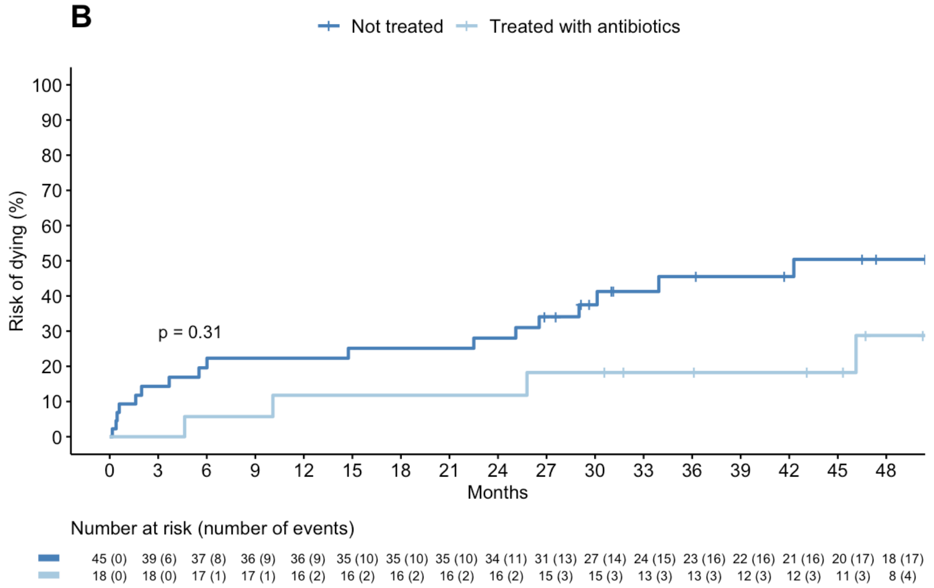

p-values are derived from the log-rank test.

Figure S3. Cumulative all-cause mortality for patients with pulmonary NTM using an imputed dataset by BACES score (Panel A) and BACES score with erythrocyte sedimentation rate replaced by C-reactive protein (> 7 mg/L) (Panel B).

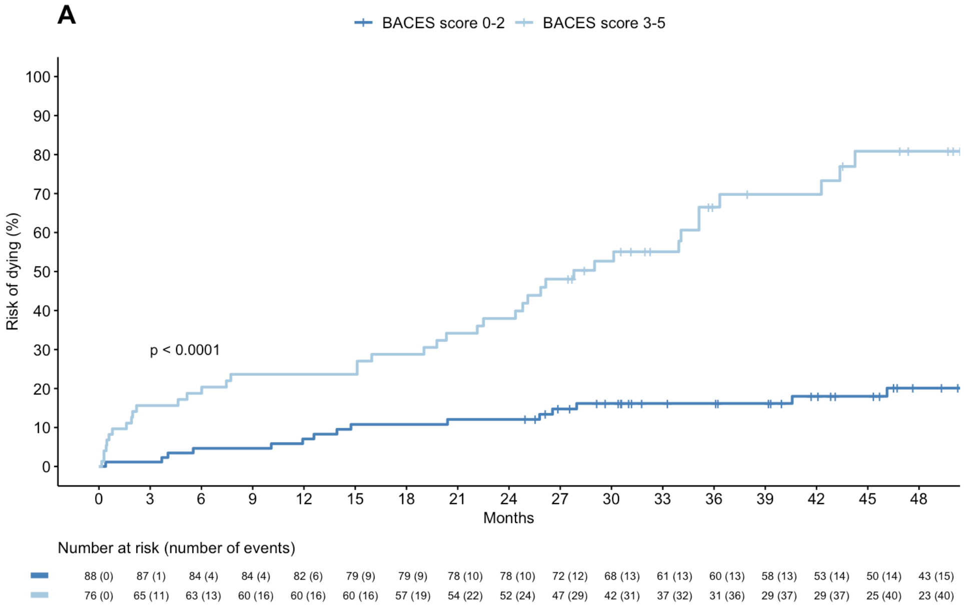

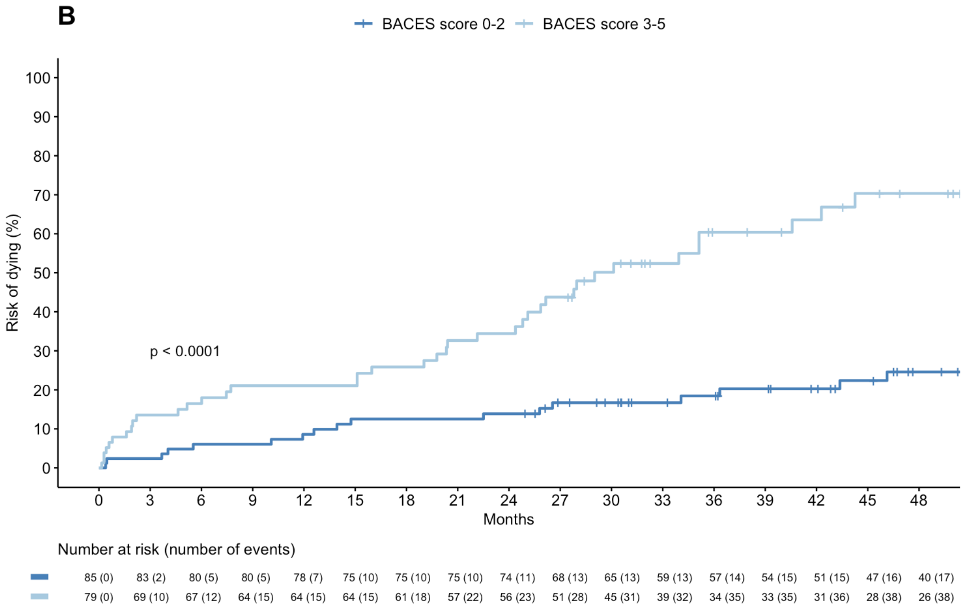

p-values are derived from the log-rank test. The BACES score is a validated score that aims to predict all-cause mortality in patients with NTM pulmonary disease (3). It is composed of body mass index <18.5 kg/m2, age ≥65 years, presence of cavity on computed tomography, elevated erythrocyte sedimentation rate (>15 mm/h in males and 20 mm/h in females), and male sex, each of which gives one point.

References

1. Hwang H, Lee JK, Heo EY, Kim DK, Lee HW. The factors associated with mortality and progressive disease of nontuberculous mycobacterial lung disease: a systematic review and meta-analysis. Sci Rep. 2023;13(1):7348.

2. Jhun BW, Moon SM, Jeon K, Kwon OJ, Yoo H, Carriere KC, et al. Prognostic factors associated with long-term mortality in 1445 patients with nontuberculous mycobacterial pulmonary disease: a 15-year follow-up study. Eur Respir J. 2020;55(1).

3. Kim HJ, Kwak N, Hong H, Kang N, Im Y, Jhun BW, et al. BACES Score for Predicting Mortality in Nontuberculous Mycobacterial Pulmonary Disease. Am J Respir Crit Care Med. 2021;203(2):230-6.

4. Sanchez-Pinto LN, Venable LR, Fahrenbach J, Churpek MM. Comparison of variable selection methods for clinical predictive modeling. Int J Med Inform. 2018;116:10-7.

5. Azur MJ, Stuart EA, Frangakis C, Leaf PJ. Multiple imputation by chained equations: what is it and how does it work? Int J Methods Psychiatr Res. 2011;20(1):40-9.

6. Yan M, Fraser B, McArthur E, Mehrabi M, Brode SK, Marras TK. External Validation of the BACES Score in Canadian Patients With Nontuberculous Mycobacterial Pulmonary Disease. Chest. 2024;165(3):521-8.

7. Fukushima K, Kitada S, Komukai S, Kuge T, Matsuki T, Kagawa H, et al. First line treatment selection modifies disease course and long-term clinical outcomes in Mycobacterium avium complex pulmonary disease. Sci Rep. 2021;11(1):1178.

8. Gochi M, Takayanagi N, Kanauchi T, Ishiguro T, Yanagisawa T, Sugita Y. Retrospective study of the predictors of mortality and radiographic deterioration in 782 patients with nodular/bronchiectatic Mycobacterium avium complex lung disease. BMJ Open. 2015;5(8):e008058.
